# Efflux Pumps and Different Genetic Contexts of *tet*(X4) Contribute to High Tigecycline Resistance in *Escherichia fergusonii* from Pigs

**DOI:** 10.3390/ijms24086923

**Published:** 2023-04-08

**Authors:** Junlin Wang, Xiulin Wan, Hecheng Meng, Rikke Heidemann Olsen, Xun Chen, Lili Li

**Affiliations:** 1 Institute of Food Safety and Nutrition, Jinan University, Guangzhou 510632, Chinacx-wolf@163.com (X.C.); 2 School of Food Science and Engineering, South China University of Technology, Guangzhou 510640, China; 3 Department of Veterinary and Animal Sciences, Faculty of Health and Medical Sciences, University of Copenhagen, 2820 Frederiksberg, Denmark

**Keywords:** *E. fergusonii*, *tet*(X4), tigecycline, efflux pump

## Abstract

Tigecycline is a last-resort antibiotic for the treatment of infections caused by multidrug-resistant bacteria. The emergence of plasmid-mediated tigecycline resistance genes is posing a serious threat to food safety and human health and has attracted worldwide attention. In this study, we characterized six tigecycline-resistant *Escherichia fergusonii* strains from porcine nasal swab samples collected from 50 swine farms in China. All the *E. fergusonii* isolates were highly resistant to tigecycline with minimal inhibitory concentration (MIC) values of 16–32 mg/L, and all contained the *tet*(X4) gene. In addition, 13–19 multiple resistance genes were identified in these isolates, revealed by whole-genome sequencing analysis. The *tet*(X4) gene was identified as being located in two different genetic structures, *hp*-*abh*-*tet*(X4)-IS*CR2* in five isolates and *hp*-*abh*-*tet*(X4)-ΔIS*CR2*-IS*Ec57*-IS*26* in one isolate. The role of efflux pumps in tigecycline resistance was evaluated by using inhibitor carbonyl cyanide 3-chlorophenylhydrazone (CCCP). The MIC values of tigecycline showed a 2- to 4-fold reduction in the presence of CCCP, indicating the involvement of active efflux pumps in tigecycline resistance in *E. fergusonii*. The *tet*(X4) gene was found to be transferable to *Escherichia coli* J53 by conjugation and resulted in the acquisition of tigcycline resistances in the transconjugants. Whole-genome multilocus sequence typing (wgMLST) and phylogenetic analysis showed a close relationship of five isolates originating from different pig farms, suggesting the transmission of *tet*(X4)-positive *E. fergusonii* between farms. In conclusion, our findings suggest that *E. fergusonii* strains in pigs are reservoirs of a transferable *tet*(X4) gene and provide insights into the tigecycline resistance mechanism as well as the diversity and complexity of the genetic context of *tet*(X4) in *E. fergusonii*.

## 1. Introduction

The emergence and spread of bacterial antimicrobial resistance pose a serious threat to food safety as well as human and animal health [1]. Of special concern is the emerging resistance to tigecycline, a tetracycline-class antibacterial agent, which has been regarded as one of the few therapeutic choices left to combat multidrug-resistant (MDR) bacterial infections [2,3].

Tigecycline resistance has emerged over recent years, mostly identified among extensively drug- and carbapenem-resistant isolates [4,5,6]. Overexpression of resistance-nodulation division (RND) efflux pumps, such as AdeABC, AdeFGH, AdeIJK, MexXY, AcrAB, TmexCD1-toprJ1 and TmexCD2-toprJ2, are important molecular mechanisms in the resistance of bacteria to tigecycline [7,8,9,10,11,12]. Currently, the global regulators of the AcrAB pump, SoxS, MarA, RamA, and Rob, have been characterized in *Enterobacteriaceae* [13], which also play a role in the decreased susceptibility to tigecycline in *Escherichia coli* and *Klebsiella* spp. [5,10,14,15,16]. Meanwhile, mutations in *plsC*, *rpsJ*, *trm*, *tet*(A), and *tet*(M) have been found to decrease tigecycline susceptibility [17,18,19,20]. In contrast, tetracycline destructases, such as Tet(X), represent a unique enzymatic tetracycline inactivation mechanism [21]. The Tet(X), a flagship tetracycline-inactivating enzyme that originated from *Bacteroides* spp. [22,23,24], has been confirmed for in vitro activity related to the degradation of all tetracyclines including tigecycline [24].

The *tet*(X4) gene is a novel plasmid-mediated high-level tigecycline resistance gene discovered in *Enterobacteriaceae* and *Acinetobacter* isolates from animals and humans in China in 2019 [2]. It was worth noting that Tet(X4) could degrade all tetracyclines [25], including tigecycline and the USFDA newly approved eravacycline [26], which poses a new threat to public health. So far, *tet*(X4) has mainly been discovered in *E. coli* from animal origin and sporadically in several other bacteria species, such as *Klebsiella pneumoniae*, *Shigella flexneri*, *Shigella boydii*, *Shigella sonnei*, *Aeromonas caviae*, *Acinetobacter* sp., and *Escherichia fergusonii* [2,18,25,27,28]. Understanding the genetic context of *tet*(X4) in different bacterial species is important to uncovering the resistance mechanism and its potential effects on human health, but this has not yet been thoroughly investigated. 

The aim of this study was to characterize six tigecycline-resistant *E. fergusonii* isolates from porcine nasal samples obtained across 50 farms in Fujian, China, including analyzing the efflux pump activity in relation to tigclycline resistance, genetic context of *tet*(X4) genes, as well as transferability and phylogenetic relationship of the strains in order to gain insight into the possible public-health impact of tigecycline-resistant *E. fergusonii* from pigs.

## 2. Results

### 2.1. Identification of E. fergusonii and Minimal Inhibitory Concentration (MIC) Values of Tigecycline

Six isolates (2022GZP175, 2022GZP221, 2022GZP331, 2022GZP462, 2022GZP491, and 2022GZP273) from six different pig farms were identified as *E. fergusonii* by 16S rRNA gene sequencing and exhibited MIC values for tigecycline between 16–32 mg/L (Table 1).

### 2.2. General Features of the E. fergusonii Genomes

In general, 13–19 acquired antimicrobial resistance genes were identified in all isolates by ResFinder, which encodes resistance to nine different antimicrobial classes, including beta-lactam, sulphonamide, aminoglycoside, disinfectant, macrolides-lincosamides-streptogramines (MLS), fluoroquinolone, trimethoprim, tetracycline, and phenicol (Table 1).

**Figure 1:**
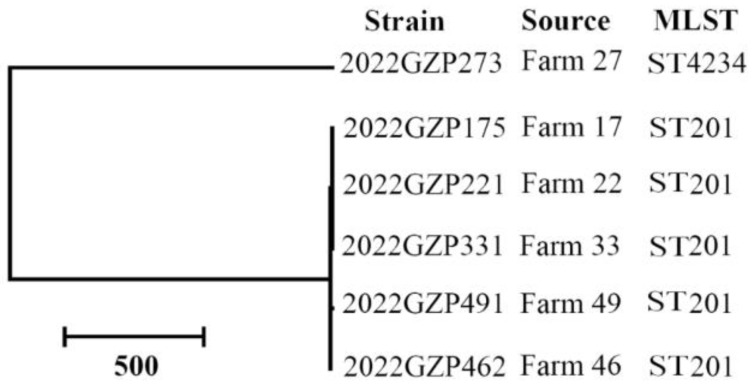
Phylogenetic analysis based on whole-genome multilocus sequence typing (wgMLST) of the six *Escherichia fergusonii* isolates.

PlasmidFinder predicted plasmid types IncFIA(HI1), IncFIB, IncHI1A, IncHI1B(R27), IncX1, and IncY in six isolates (Table 1). MLST typing identified two types in the six isolates, in which five isolates were the same MLST type (ST201) and one isolate was ST4234 (Table 1, Figure 1).

### 2.3. Genetic Context of Tigecycline Resistance Gene

**Figure 2:**
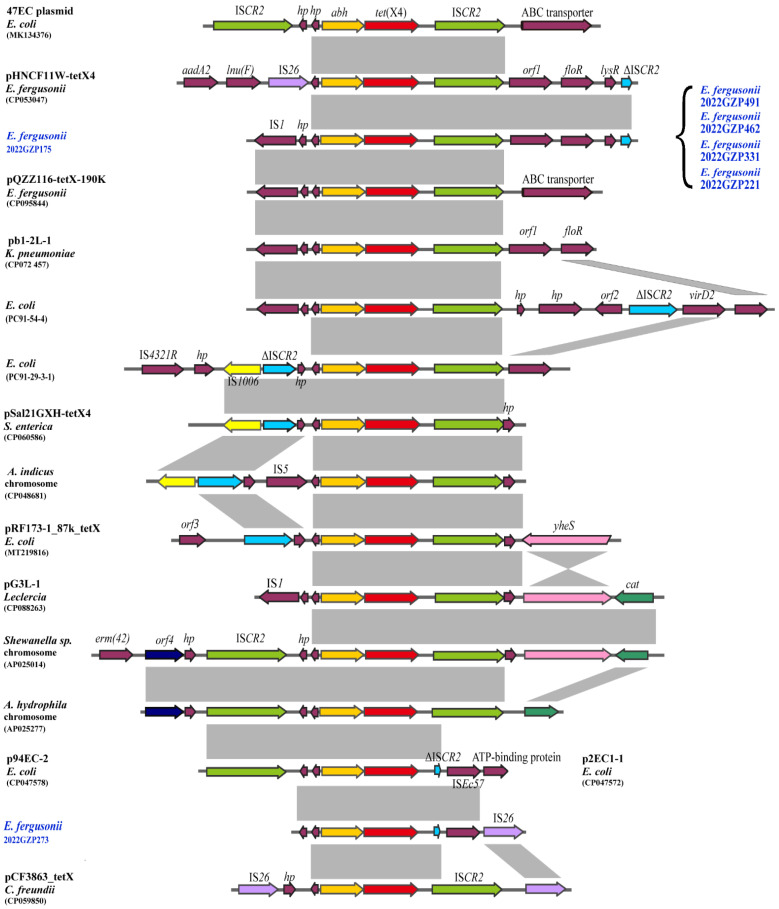
Genetic environments of the *tet*(X4) gene in six *Escherichia fergusonii* isolates isolated from 50 pig farms in China. The arrows indicate open reading frames. Light-gray shading denotes homology regions. Strains in this study are in blue font.

All strains contained the *tet*(X4) gene. In addition to *tet*(X4), *tet*(B) without mutation was identified in isolate 2022GZP273, and *tet*(A), as well as *tet*(M) with the same mutations, was found in all the other isolates (Table 1 and Table S1). For five of the six isolates, the *tet*(X4) gene was located in a classic genetic context, *hp*-*abh*-*tet*(X4)-IS*CR2*, while a new genetic context of *tet*(X4), *hp*-*abh*-*tet*(X4)-ΔIS*CR2*-IS*Ec57*-IS*26*, was observed in isolate 2022GZP273 (Figure 2).

The classic genetic context, *hp*-*abh*-*tet*(X4)-IS*CR2*, has been mainly identified in plasmids in *E. coli* and less frequently in other bacteria species (Figure 2, Supplementary Table S2). Two *E. fergusonii* isolates have previously been reported to be hosts of this structure, as indicated in Figure 2 [29,30]. Compared with the reported genetic context in the two *E. fergusonii* isolates, *hp*-*abh*-*tet*(X4)-IS*CR2* share the same downstream structure as those in pHNCF11W-tetX4 (GenBank accession number CP053047), and the same upstream structure as pQZZ116-tetX-190K (GenBank accession number CP095844) (Figure 2).

In the new genetic context, *hp*-*abh*-*tet*(X4)-ΔIS*CR2*-IS*Ec57*-IS*26*, IS*CR2* was truncated by IS*Ec57* and linked by a copy of IS*26* (Figure 2). In the analysis, two *E. coli* isolates, obtained from a human gut in Singapore in 2019, were identified as containing *hp*-*abh*-*tet*(X4)-ΔIS*CR2*-IS*Ec57* without IS*26* (GenBank accession numbers CP047578 and CP047572) (Figure 2).

### 2.4. Efflux Pumps’ Activity

The MIC value of CCCP is 8 mg/L for all isolates. After addition of 2 mg/L CCCP, there was a fourfold decline in the MIC value for the tigecycline of one isolate (2022GZP491) and half a decline in the MIC values for the tigecycline of the remaining five isolates (2022GZP462, 2022GZP331, 2022GZP221, 2022GZP175 and 2022GZP273), indicating the presence of active efflux pumps mediating tigecycline resistance in all isolates (Table 1).

The known efflux pumps contributing to tigecycline resistance, AcrAB-TolC, AcrZ and NorM, were identified in the genome sequences of all isolates. In addition to the known efflux pumps, several efflux pumps and porins associated with multi-drug resistance, such as OmpF outer membrane porin, MarA and its local repressor MarR, were also identified in all isolates (Table S3).

### 2.5. Conjugation

Polymerase Chain Reaction (PCR) results confirmed the successful transfer of the *tet*(X4) genes from all *E*. *fergusonii* strains to a plasmid-free recipient *E. coli* J53. Antimicrobial susceptibility testing revealed that the acquisition of the *tet*(X4) genes by *E. coli* J53 caused at least a 32-fold increase for tigecycline (Table 2). The conjugation rates ranged from 3.4 × 10^−7^ to 2.6 × 10^−6^ transconjugant per recipient cell in *E*. *fergusonii* strains (Table 2).

### 2.6. Phylogenetic Analysis

The wgMLST and phylogenetic analysis showed that isolates 2022GZP462, 2022GZP331, 2022GZP221, 2022GZP175, and 2022GZP491 were all highly related, while they were distantly related to 2022GZP273 (Figure 1).

## 3. Discussion

*E. fergusonii* is an opportunistic pathogen infecting humans and animals [31]. It causes a wide range of infections in poultry and has incurred significant economic losses worldwide [32], and it has been reported in several clinical cases in humans, including wound infections, urinary tract infections, bacteremia, and diarrhoea [33]. *E. fergusonii* from livestock have been reported to be an underrated repository for antimicrobial resistance, especially with regards to *mcr*-*1* gene [34]. Thus, the emergence of tigecycline resistance in *E. fergusonii* significantly increases its importance to public health [29,34].

Tigecycline resistance has been found in many species. However, tigecycline-resistant *E. fergusonii* isolates have only been reported in two samples in China, a pig feces sample and a chicken feces sample [34,35]. In these two isolates, the tigecycline resistance was found to be associated with the *tet*(X4) gene, which was located in the classic genetic structure *hp*-*abh*-*tet*(X4)-IS*CR2*. Similar to these findings, for five out of six *E. fergusonii* isolates in the present study, the *tet*(X4) gene was located in *hp*-*abh*-*tet*(X4)-IS*CR2*. Interestingly, the genetic structure of *tet*(X4) in these *E. fergusonii* isolates shared either the same upstream or downstream sequences, indicating that a recombination process at an earlier stage is likely to have happened at fixed sites in *E. fergusonii.* Importantly, the wide distribution of the *hp*-*abh*-*tet*(X4)-IS*CR2* in broad bacterial species indicates its high transferability, which has also been observed in this study and which might cause an expansion of tigecycline resistance.

Notably, a novel genetic structure, *hp*-*abh*-*tet*(X4)-ΔIS*CR2*-IS*Ec57*-IS*26*, was observed in an *E. fergusonii* isolate. In this structure, the downstream IS*CR2* was truncated and inserted by IS*Ec57* and was associated with IS*26*. IS*CR2* is likely to have been truncated by different IS elements, such as IS*26*, IS*1D*, IS*1R*, IS*Ec57*, and IS*Kpn19* [18]. However, the unit *hp*-*abh*-*tet*(X4)-ΔIS*CR2*-IS*Ec57* has only been observed in two *E. coli* isolates. Notably, the unit was found to be associated with one more IS*26* in the *E. fergusonii* isolate in this study, suggesting that the emergence of this structure in *E. fergusonii* was a newer event at a molecular level. IS*26* can mediate the formation of a hybrid plasmid between *tet*(X4)-positive and -negative plasmids [36]. Thus, the combined effect of IS*CR2*, IS*Ec57* and IS*26* might be a major driving force in the rapid expansion of *tet*(X4) in *E. fergusonii*. 

Besides the *tet*(X4) gene, efflux pumps were observed to be a contributor to tigecycline resistance in *E. fergusonii* isolates in the current study. The contribution of efflux pumps to tigecycline resistance in different bacterial species has been investigated in many studies [20,37,38]. However, the activity of efflux pumps on tigecycline resistance in *E. fergusonii* has not been elucidated. In this study, by searching through genome sequences, we identified various RND-type efflux pumps (AcrAB-TolC, AcrZ and NorM) that existed in all *E. fergusonii* isolates, which have been confirmed to be associated with tigecycline resistance [14,38,39]. The contribution of efflux pumps to tigecycline resistance in *E. fergusonii* was further revealed by the efflux pump inhibitor, CCCP. CCCP has been shown to have a good activity against RND-type efflux pumps associated with tigecycline resistance [37]. In this study, the addition of CCCP resulted in a 2- to 4-fold decline in MIC values for tigecycline, indicating that RND-type efflux pumps that exist in *E. fergusonii* isolates also partly contribute to the high level of tigecycline resistance. However, in addition to *tet*(X4) and RND-type efflux pumps, which were confirmed to contribute to tigecycline resistance in *E. fergusonii*, we cannot exclude the fact that other factors may also contribute to phenotypical tigclycline resistance, such as mutations in *tet*(A) and *tet*(M) genes, OmpF outer membrane porin, MarA and its local repressor MarR, which have been described as contributing to overexpression of the AcrAB efflux pump and may indirectly lead to phenotypical tigclycline resistance in *E. fergusonii* [40,41,42,43]. Therefore, more in vitro research is needed to determine how these different mutation types and factors are involved in tigclycline resistance in *E. fergusonii*.

Unexpectedly, the five *E. fergusonii* isolates harboring the same genetic structure of *tet*(X4) were closely related, despite being obtained from different farms. This finding could indicate that *E. fergusonii* in pigs are reservoirs of the *tet*(X4) gene and that *tet*(X4)-positive *E. fergusonii* may have been transmitted between different pig farms in China. Since *E. fergusonii* isolates in this study were all multi-drug-resistant, the emergence of transferable *tet*(X4) in *E. fergusonii* from pigs needs to be monitored and further investigated, as it may spread along the food chain to humans.

## 4. Materials and Methods

### 4.1. Bacterial Isolation and Identification

In August 2019, a total of 250 porcine nasal swab samples were collected from a total of 50 farms in Fujian, China. Five samples were collected from each farm and pooled for strain isolation. A low-temperature box with ice was used to transport samples from the field to the laboratory for further processing. The samples were incubated in buffered peptone water (BPW) broth for 18 to 24 h and then inoculated onto Luria–Bertani (LB; Guangdong Huankai Microbial Sci. & Tech., Guangzhou, China) agar plates with 2 mg/L tigecycline. Colonies were identified using 16S rRNA gene sequencing using universal primers (16S-F, 5′-AGAGTTTGATCCTGG CTCAG-3′; 16S-R, 5′-GGTTACCTTGTTACGACTT-3′) [44].

### 4.2. Antimicrobial Susceptibility Testing

MIC of tigecycline (Sigma-Aldrich, St. Louis, MO, USA) was determined by broth microdilution [45]. The resistance breakpoint (>2 mg/L) was interpreted as resistant according to the European Committee on Antimicrobial Susceptibility Testing (EUCAST) guidelines (http://www.eucast.org/clinical_breakpoints/, (accessed on 5 March 2021). *E. coli* ATCC25922 was used as a control, and all experiments were performed with three biological replicates.

### 4.3. Whole-Genome Sequencing and Annotation

The whole genome of a total of six *E. fergusonii* isolates was sequenced on Illumina Novaseq-PE150 150-bp paired-end reads (Personal Biotechnology Co., Shanghai, China). The initial data quality inspection was performed with FastQC (v0.11.9, https://www.bioinformatics.babraham.ac.uk./projects/fastqc, (accessed on 15 May 2022), after which reads were filtered and trimmed using Cutadapt (v1.17) to discard the low-quality reads that contained ambiguous nucleotides or a quality score lower than 20 [46]. The genome was assembled by EToKi modules in Enterobase (https://enterobase.warwick.ac.uk/, (accessed on 20 May 2022) [47]. The presence of acquired antibiotic resistance genes and plasmids was assessed by ResFinder [48] and further determined by BLASTn2 (http://blast.ncbi.nlm.nih.gov/Blast.cgi, (accessed on 21 May 2022). Clonal analysis was assessed by MLST 2.0 (https://enterobase.warwick.ac.uk/species/senterica/allele_st_search, (accessed on 21 May 2022). PlasmidFinder V2.1 was used to identify plasmid replicon types [49].

### 4.4. Effect of CCCP on Tigecycline MIC

The activity of the efflux pump system on tigecycline MIC was tested using efflux pump inhibitor CCCP [37]. The MIC of CCCP for each isolate was tested first, and then a final concentration of 1/4 MIC (2 mg/L) of CCCP (subinhibitory concentrations that did not affect bacterial growth) was added to each well when testing MIC for tigecycline.

### 4.5. Phylogenetic Analysis of the Genomic Sequences

In order to assess the relatedness of the six *E. fergusonii* isolates, a minimum spanning tree was constructed in Enterobase using the RapidNJ algorithm and the whole genome multilocus sequence typing (wgMLST) (wgMLST scheme available on EnteroBase) scheme [50].

### 4.6. Conjugation Experiments

The transferability of *tet*(X4) was assessed by performing the conjugation experiment, using solid mating on a filter (Whatman, Maidstone, UK). The sodium azide-resistant *E. coli* strain J53 was used as a recipient strain [51].

Briefly, recipient and donor strains were cultured overnight in LB broth, and then the cells were harvested, washed with saline, mixed together in a ratio of 1:1, and spotted onto a 0.45 µm pore size filter (Millipore) on LB plates to be cultured for 20 h. They were also spotted individually on LB plates as controls. The transconjugants were selected on LB plates containing 150 mg/L sodium azide and 2, 4, 8, or 16 mg/L of tigecycline after being cultured for 24–48 h. The test was conducted at least three times. Control spots were transferred to the same selective media to make sure that no growth was observed.

The conjugation frequency was calculated as the ratio of transconjugants over the number of recipients. The transfer of the plasmid was confirmed by PCR, with the primers listed in Table S4.

### 4.7. Nucleotide Sequence Accession Numbers

The Illumina sequence data were deposited in the Enterobase database under the barcode numbers ESC_ZA4185AA, ESC_ZA4186AA, ESC_ZA4187AA, ESC_ZA4188AA, ESC_ZA4189AA, and ESC_ZA4190AA.

## 5. Conclusions

To summarize, this study for the first time reports the involvement of efflux pumps and transferable diverse genetic structures of *tet*(X4) in the tigecycline resistance of *E. fergusonii.* Our study revealed that *E. fergusonii* in pigs are reservoirs of *tet*(X4) gene and that they may have been transmitted between different pig farms in China, which poses potential hazards to associated pork products’ safety and a public health risk and which thus requires continuous investigations.

## Figures and Tables

**Table 1:** The antibiotic susceptibility of tigecycline, effect of carbonyl cyanide 3-chlorophenylhydrazone (CCCP) on MIC values of tigecycline, and predicted plasmids of *E. fergusonii* isolates.

Strain	MIC (mg/L)	MIC + CCCP (mg/L)	Plasmid Inc Group	TIG ^a^	Other Resistance Determinants	Antibiotic Resistance ^b^
2022GZP273	16	8	IncFIA(HI1), IncFIB, IncHI1A, IncHI1B(R27), IncX1, IncY	*tet*(X4)	*aadA1*, *aadA2*, *aadA22*, *aadA24*, *aph(3′)*-*Ia*, *aph(3″)*-*Ib*, *aph(6)*-*Id*, *blaTEM*-*1B*, *cmlA1*, *dfrA12*, *erm(42)*, *floR*, *lnu(G)*, *mef(B)*, *qacL*, *qnrS1*, *qnrS2*, *sul3*, *tet*(B)	AMIs, BETs, DISs, FLUs, MLS, PHEs, SULs, TETs, TRIs
2022GZP491	32	8	IncX1, IncY	*tet*(X4)	*aadA1*, *aadA2*, *blaTEM*-*1B*, *cmlA1*, *dfrA12*, *erm(42)*, *floR*, *qacL*, *qnrS1*, *sul2*, *sul3*, *tet*(A), *tet*(M)	AMIs, BETs, DISs, FLUs, MLS, PHEs, SULs, TETs, TRIs
2022GZP462	32	16	IncX1, IncY	*tet*(X4)	*aadA1*, *aadA2*, *blaTEM*-*1B*, *cmlA1*, *dfrA12*, *erm(42)*, *floR*, *qacL*, *qnrS1*, *sul2*, *sul3*, *tet*(A), *tet*(M)	AMIs, BETs, DISs, FLUs, MLS, PHEs, SULs, TETs, TRIs
2022GZP331	32	16	IncFIB, IncX1, IncY	*tet*(X4)	*aadA1*, *aadA2*, *blaTEM*-*1B*, *cmlA1*, *dfrA12*, *erm(42)*, *floR*, *qacL*, *qnrS1*, *sul2*, *sul3*, *tet*(A), *tet*(M)	AMIs, BETs, DISs, FLUs, MLS, PHEs, SULs, TETs, TRIs
2022GZP221	16	8	IncFIB, IncX1, IncY	*tet*(X4)	*aadA1*, *aadA2*, *blaTEM*-*1B*, *cmlA1*, *dfrA12*, *erm(42)*, *floR*, *qacL*, *qnrS1*, *sul2*, *sul3*, *tet*(A), *tet*(M)	AMIs, BETs, DISs, FLUs, MLS, PHEs, SULs, TETs, TRIs
2022GZP175	32	16	IncFIB, IncX1, IncY	*tet*(X4)	*aadA1*, *aadA2*, *blaTEM*-*1B*, *cmlA1*, *dfrA12*, *erm(42)*, *floR*, *qacL*, *qnrS1*, *sul2*, *sul3*, *tet*(A), *tet*(M)	AMIs, BETs, DISs, FLUs, MLS, PHEs, SULs, TETs, TRIs

^a^ TIG, tigcycline resistance gene. ^b^ AMIs, aminoglycosides; BETs, beta-lactams; DISs, disinfectants; FLUs, fluoroquinolones; MLS, macrolide, lincosamide and streptogramin B; PHEs, phenicols; SULs, sulphonamides; TETs, tetracyclines; TRIs, trimethoprims.

**Table 2:** MICs of *E. fergusonii* strains, *E. coli* J53 and selected transconjugants.

Strain	MIC of Tigcycline (mg/L)	Conjugation Rates
*E. coli* J53	0.25	
2022GZP273	16	
2022GZP491	32	
2022GZP462	32	
2022GZP331	32	
2022GZP221	16	
2022GZP175	32	
2022GZP273 transconjugant	8	2.6 × 10^−6^ ± 0.4
2022GZP491 transconjugant	8	5.5 × 10^−7^ ± 0.5
2022GZP462 transconjugant	8	3.4 × 10^−7^ ± 0.3
2022GZP331 transconjugant	8	6.7 × 10^−7^ ± 0.4
2022GZP221 transconjugant	8	5.3 × 10^−7^ ± 0.6
2022GZP175 transconjugant	8	7.4 × 10^−7^ ± 0.6

## Data Availability

Data are contained within the article or Supplementary Material.

## Supplementary Materials

The supporting information can be downloaded at: https://www.mdpi.com/article/10.3390/ijms24086923/s1. References [52,53] are cited in the supplementary materials.

## References

[1] Laxminarayan R., Sridhar D., Blaser M., Wang M., Woolhouse M. (2016). Achieving global targets for antimicrobial resistance. Science.

[2] He T., Wang R., Liu D., Walsh T.R., Zhang R., Lv Y., Ke Y., Ji Q., Wei R., Liu Z. (2019). Emergence of plasmid-mediated high-level tigecycline resistance genes in animals and humans. Nat. Microbiol..

[3] Sun Y., Cai Y., Liu X., Bai N., Liang B., Wang R. (2013). The emergence of clinical resistance to tigecycline. Int. J. Antimicrob. Agents.

[4] Pournaras S., Koumaki V., Gennimata V., Kouskouni E., Tsakris A. (2015). In vitro activity of tigecycline against *Acinetobacter baumannii*: Global epidemiology and resistance mechanisms. Advances in Microbiology, Infectious Diseases and Public Health.

[5] Pournaras S., Koumaki V., Spanakis N., Gennimata V., Tsakris A. (2016). Current perspectives on tigecycline resistance in Enterobacteriaceae: Susceptibility testing issues and mechanisms of resistance. Int. J. Antimicrob. Agents.

[6] Wu J., Sun L., Chen X., Du F., Shi H., Chen C., Chen Z.J. (2013). Cyclic GMP-AMP is an endogenous second messenger in innate immune signaling by cytosolic DNA. Science.

[7] Hentschke M., Christner M., Sobottka I., Aepfelbacher M., Rohde H. (2010). Combined *ramR* mutation and presence of a Tn*1721*-associated *tet*(A) variant in a clinical isolate of *Salmonella enterica* serovar Hadar resistant to tigecycline. Antimicrob. Agents Chemother..

[8] Keeney D., Ruzin A., Bradford P.A. (2007). RamA, a transcriptional regulator, and AcrAB, an RND-type efflux pump, are associated with decreased susceptibility to tigecycline in *Enterobacter cloacae*. Microb. Drug. Resist..

[9] Lv L., Wan M., Wang C., Gao X., Yang Q., Partridge S.R., Wang Y., Zong Z., Doi Y., Shen J. (2020). Emergence of a plasmid-encoded resistance-nodulation-division efflux pump conferring resistance to multiple drugs, including tigecycline, in *Klebsiella pneumoniae*. mBio.

[10] Veleba M., De Majumdar S., Hornsey M., Woodford N., Schneiders T. (2013). Genetic characterization of tigecycline resistance in clinical isolates of *Enterobacter cloacae* and *Enterobacter aerogenes*. J. Antimicrob. Chemother..

[11] Wang C.Z., Gao X., Yang Q.W., Lv L.C., Wan M., Yang J., Cai Z.P., Liu J.H. (2021). A novel transferable resistance-nodulation-division pump gene cluster, *tmexCD2-toprJ2*, confers tigecycline resistance in Raoultella ornithinolytica. Antimicrob. Agents Chemother..

[12] Xu J., Zhu Z., Chen Y., Wang W., He F. (2021). The plasmid-borne *tet*(A) gene is an important factor causing tigecycline resistance in ST11 carbapenem-resistant *Klebsiella pneumoniae* under selective pressure. Front. Microbiol..

[13] Wang X., Chen H., Zhang Y., Wang Q., Zhao C., Li H., He W., Zhang F., Wang Z., Li S. (2015). Genetic characterisation of clinical *Klebsiella pneumoniae* isolates with reduced susceptibility to tigecycline: Role of the global regulator RamA and its local repressor RamR. Int. J. Antimicrob. Agents.

[14] He F., Fu Y., Chen Q., Ruan Z., Hua X., Zhou H., Yu Y. (2015). Tigecycline susceptibility and the role of efflux pumps in tigecycline resistance in KPC-producing *Klebsiella pneumoniae*. PLoS ONE.

[15] Pérez A., Poza M., Aranda J., Latasa C., Medrano F.J., Tomás M., Romero A., Lasa I., Bou G. (2012). Effect of transcriptional activators SoxS, RobA, and RamA on expression of multidrug efflux pump AcrAB-TolC in *Enterobacter cloacae*. Antimicrob. Agents Chemother..

[16] Ruzin A., Keeney D., Bradford P.A. (2005). AcrAB efflux pump plays a role in decreased susceptibility to tigecycline in *Morganella morganii*. Antimicrob. Agents Chemother..

[17] Haim M.S., Di Gregorio S., Galanternik L., Lubovich S., Vázquez M., Bharat A., Zaheer R., Golding G.R., Graham M., Van Domselaar G. (2017). First description of *rpsJ* and *mepA* mutations associated with tigecycline resistance in *Staphylococcus aureus* isolated from a cystic fibrosis patient during antibiotic therapy. Int. J. Antimicrob. Agents.

[18] Liu D., Wang T., Shao D., Song H., Zhai W., Sun C., Zhang Y., Zhang M., Fu Y., Zhang R. (2022). Structural diversity of the ISCR2-mediated rolling-cycle transferable unit carrying *tet*(X4). Sci. Total Environ..

[19] Niebel M., Quick J., Prieto A.M., Hill R.L., Pike R., Huber D., David M., Hornsey M., Wareham D., Oppenheim B. (2015). Deletions in a ribosomal protein-coding gene are associated with tigecycline resistance in *Enterococcus faecium*. Int. J. Antimicrob. Agents.

[20] Yang Y.S., Chen H.Y., Hsu W.J., Chou Y.C., Perng C.L., Shang H.S., Hsiao Y.T., Sun J.R., Chang Y.Y., Liu Y.M. (2019). Overexpression of AdeABC efflux pump associated with tigecycline resistance in clinical *Acinetobacter nosocomialis* isolates. Clin. Microbiol. Infect..

[21] Linkevicius M., Sandegren L., Andersson D.I. (2016). Potential of tetracycline resistance proteins to evolve tigecycline resistance. Antimicrob. Agents Chemother..

[22] Deng M., Zhu M.H., Li J.J., Bi S., Sheng Z.K., Hu F.S., Zhang J.J., Chen W., Xue X.W., Sheng J.F. (2014). Molecular epidemiology and mechanisms of tigecycline resistance in clinical isolates of *Acinetobacter baumannii* from a Chinese university hospital. Antimicrob. Agents Chemother..

[23] Leski T.A., Bangura U., Jimmy D.H., Ansumana R., Lizewski S.E., Stenger D.A., Taitt C.R., Vora G.J. (2013). Multidrug-resistant *tet*(X)-containing hospital isolates in Sierra Leone. Int. J. Antimicrob. Agents.

[24] Moore I.F., Hughes D.W., Wright G.D. (2005). Tigecycline is modified by the flavin-dependent monooxygenase TetX. Biochemistry.

[25] Martelli F., AbuOun M., Cawthraw S., Storey N., Turner O., Ellington M., Nair S., Painset A., Teale C., Anjum M.F. (2022). Detection of the transferable tigecycline resistance gene *tet*(X4) in *Escherichia coli* from pigs in the United Kingdom. J. Antimicrob. Chemother..

[26] Sun J., Chen C., Cui C.Y., Zhang Y., Liu X., Cui Z.H., Ma X.Y., Feng Y., Fang L.X., Lian X.L. (2019). Plasmid-encoded *tet*(X) genes that confer high-level tigecycline resistance in *Escherichia coli*. Nat. Microbiol..

[27] Liu Y.Y., Wang Y., Walsh T.R., Yi L.X., Zhang R., Spencer J., Doi Y., Tian G., Dong B., Huang X. (2016). Emergence of plasmid-mediated colistin resistance mechanism MCR-1 in animals and human beings in China: A microbiological and molecular biological study. Lancet. Infect. Dis..

[28] Yong D., Toleman M.A., Giske C.G., Cho H.S., Sundman K., Lee K., Walsh T.R. (2009). Characterization of a new metallo-β-lactamase gene, *bla_NDM-1_*, and a novel erythromycin esterase gene carried on a unique genetic structure in *Klebsiella pneumoniae* sequence type 14 from India. Antimicrob. Agents Chemother..

[29] Guan C., Tang B., Yang H., Ma J., Huang Y., Liu C. (2022). Emergence of plasmid-mediated tigecycline resistance gene, *tet*(X4), in *Escherichia fergusonii* from pigs. J. Glob. Antimicrob. Resist..

[30] Li Y., Wang Q., Peng K., Liu Y., Li R., Wang Z. (2020). Emergence of carbapenem-and tigecycline-resistant *Proteus cibarius* of animal origin. Front. Microbiol..

[31] Farmer J.J., Fanning G.R., Davis B.R., O’Hara C.M., Riddle C., Hickman-Brenner F.W., Asbury M.A., Lowery V.A., Brenner D.J. (1985). *Escherichia fergusonii* and *Enterobacter taylorae*, two new species of Enterobacteriaceae isolated from clinical specimens. J. Clin. Microbiol..

[32] Saha O., Rakhi N.N., Hoque M.N., Sultana M., Hossain M.A. (2021). Genome-wide genetic marker analysis and genotyping of *Escherichia fergusonii* strain OTSVEF-60. Braz. J. Microbiol..

[33] Lagacé-Wiens P.R., Baudry P.J., Pang P., Hammond G. (2010). First description of an extended-spectrum-β-lactamase-producing multidrug-resistant *Escherichia fergusonii* strain in a patient with cystitis. J. Clin. Microbiol..

[34] Tang B., Chang J., Chen Y., Lin J., Xiao X., Xia X., Lin J., Yang H., Zhao G. (2022). *Escherichia fergusonii*, an underrated repository for antimicrobial resistance in food animals. Microbiol. Spectrum..

[35] Li R., Lu X., Munir A., Abdullah S., Liu Y., Xiao X., Wang Z., Mohsin M. (2022). Widespread prevalence and molecular epidemiology of *tet*(X4) and *mcr*-*1* harboring *Escherichia coli* isolated from chickens in Pakistan. Sci. Total Environ..

[36] Li R., Peng K., Li Y., Liu Y., Wang Z. (2020). Exploring *tet*(X)-bearing tigecycline-resistant bacteria of swine farming environments. Sci. Total Environ..

[37] Ardehali S.H., Azimi T., Fallah F., Owrang M., Aghamohammadi N., Azimi L. (2019). Role of efflux pumps in reduced susceptibility to tigecycline in *Acinetobacter baumannii*. New Microbes New Infect..

[38] Hobbs E.C., Yin X., Paul B.J., Astarita J.L., Storz G. (2012). Conserved small protein associates with the multidrug efflux pump AcrB and differentially affects antibiotic resistance. Proc. Natl. Acad. Sci. USA.

[39] Morita Y., Kodama K., Shiota S., Mine T., Kataoka A., Mizushima T., Tsuchiya T. (1998). NorM, a putative multidrug efflux protein, of *Vibrio parahaemolyticus* and its homolog in *Escherichia coli*. Antimicrob. Agents Chemother..

[40] Alekshun M.N., Levy S.B. (1997). Regulation of chromosomally mediated multiple antibiotic resistance: The *mar* regulon. Antimicrob. Agents Chemother..

[41] Barbosa T.M., Levy S.B. (2000). Differential expression of over 60 chromosomal genes in *Escherichia coli* by constitutive expression of MarA. J. Bacteriol..

[42] Chollet R., Chevalier J., Bollet C., Pages J.M., Davin-Regli A. (2004). RamA is an alternate activator of the multidrug resistance cascade in *Enterobacter aerogenes*. Antimicrob. Agents Chemother..

[43] Keeney D., Ruzin A., McAleese F., Murphy E., Bradford P.A. (2007). MarA-mediated overexpression of the AcrAB efflux pump results in decreased susceptibility to tigecycline in *Escherichia coli*. J. Antimicrob. Chemother..

[44] Li L., Olsen R.H., Wang C., Song A., Xiao J., Meng H., Ronco T., Shi L. (2021). First report of two foodborne *Salmonella enterica* subsp. *enterica* serovar Bovismorbificans isolates carrying a novel mega-plasmid harboring *bla_DHA-1_* and *qnrB4* genes. Int. J. Food Microbiol..

[45] CLSI (2021). Performance Standards for Antimicrobial Susceptibility Testing.

[46] Martin M. (2011). Cutadapt removes adapter sequences from high-throughput sequencing reads. EMBnet J..

[47] Jagadeesan B., Baert L., Wiedmann M., Orsi R.H. (2019). Comparative analysis of tools and approaches for source tracking *Listeria monocytogenes* in a food facility using whole-genome sequence data. Front. Microbiol..

[48] Zankari E., Hasman H., Cosentino S., Vestergaard M., Rasmussen S., Lund O., Aarestrup F.M., Larsen M.V. (2012). Identification of acquired antimicrobial resistance genes. J. Antimicrob. Chemother..

[49] Carattoli A., Hasman H. (2020). PlasmidFinder and In Silico pMLST: Identification and Typing of Plasmid Replicons in Whole-Genome Sequencing (WGS). Methods Mol. Biol..

[50] Zhou Z., Alikhan N.F., Mohamed K., Fan Y., Achtman M., Agama Study Group (2020). The EnteroBase user’s guide, with case studies on *Salmonella* transmissions, *Yersinia pestis* phylogeny, and *Escherichia* core genomic diversity. Genome Res..

[51] Hammerum A.M., Hansen F., Nielsen H.L., Jakobsen L., Stegger M., Andersen P.S., Jensen P., Nielsen T.K., Hansen L.H., Hasman H. (2016). Use of WGS data for investigation of a long-term NDM-1-producing *Citrobacter freundii* outbreak and secondary in vivo spread of *bla_NDM-1_* to *Escherichia coli*, *Klebsiella pneumoniae* and *Klebsiella oxytoca*. J. Antimicrob. Chemother..

[52] Lindsey R.L., Garcia-Toledo L., Fasulo D., Gladney L.M., Strockbine N. (2017). Multiplex polymerase chain reaction for identification of *Escherichia coli*, *Escherichia albertii* and *Escherichia fergusonii*. J. Microbiol. Methods.

[53] Ji K., Xu Y., Sun J., Huang M., Jia X., Jiang C., Feng Y. (2020). Harnessing efficient multiplex PCR methods to detect the expanding *tet*(X) family of tigecycline resistance genes. Virulence.

