# Supplementary material for: Efflux Pumps and Different Genetic Contexts of tet(X4) Contribute to High Tigecycline Resistance in Escherichia fergusonii from Pigs
Source: Int J Mol Sci. 2023 Apr 8;24(8):6923. doi: 10.3390/ijms24086923 (PMC10138661; doi:10.3390/ijms24086923)
Supplement: Supplementary file 1 [file ijms-24-06923-s001.zip › ijms-2297244-supplementary.pdf]

Supplementary Data

Table S1. The identity of *tet* genes in six *E. fergusonii* isolates.

Strains	Resistance gene	Identity (%)	Coverage (%)	Position in contig	Accession no. of reference gene
2022GZP273	*tet*(A)	99.9167	100	1177..2376	AJ517790
	tet(M)	96.1458	100	973..2892	X04388
2022GZP491	*tet*(A)	99.9167	100	1576..2775	AJ517790
	tet(M)	96.1458	100	973..2892	X04388
2022GZP462	*tet*(A)	99.9167	100	1576..2775	AJ517790
	tet(M)	96.1458	100	973..2892	X04388
2022GZP331	*tet*(A)	99.9167	100	1177..2376	AJ517790
	tet(M)	96.1458	100	448..2367	X04388
2022GZP221	*tet*(A)	99.9167	100	1576..2775	AJ517790
	tet(M)	96.1458	100	973..2892	X04388
2022GZP175	*tet*(B)	100	100	4159..5364	AF326777

Table S2. Sequences with 97% of coverage and 98% identity with module *hp-abh-tet*(X4)-IS*CR2* according to Blastn alignment using NCBI GenBank database.

Taxonomy	Number of sequence of 98% identity matches	Number of Organisms
Gammaproteobacteria	136	22
. Enterobacterales	128	17
.. Enterobacteriaceae	124	14
... Escherichia	101	3
.... Escherichia coli	90	1
.... Escherichia fergusonii	2	1
.... Escherichia sp.	9	1
... Klebsiella	12	4
.... Klebsiella pneumoniae	6	1
.... Klebsiella quasipneumoniae	2	1
.... Klebsiella sp.	3	1
.... Klebsiella aerogenes	1	1
... Enterobacter cloacae complex	3	2

Table S3. Multidrug efflux pump genes, multidrug efflux pump regulatory genes, and porins genes of isolates.

Strain	Multidrug efflux pump genes and porins genes
2022GZP273	*AcrAB-TolC*, *AcrZ*, *norM*, *ompA*, *ompC*, *ompF*, *ompR*, *ompX*, *ompN_1*, *ompN_2*, *rob*, *marA*, *marB*, *marC*, *marR*, *emrA*, *emrB*, *emrE*, *mdfA*, *acrD*, *mexE*, *(Bcr/CflA)*
2022GZP491	*AcrAB-TolC*, *AcrZ*, *norM*, *ompA*, *ompC*, *ompF*, *ompG*, *ompR*, *ompW*, *ompX*, *ompN_1*, *ompN_2*, *soxS*, *rob*, *marA*, *marB*, *marC*, *marR*, *emrA*, *emrB*, *emrE*, *mdfA*, *mexE*, *acrD*, *mexE*, *(Bcr/CflA)*
2022GZP462	*AcrAB-TolC*, *AcrZ*, *norM*, *ompC*, *ompF*, *ompG*, *ompR*, *ompW*, *ompX*, *ompN_1*, *ompN_2*, *soxS*, *rob*, *marA*, *marB*, *marC*, *marR*, *emrA*, *emrB*, *emrE*, *mdfA*, *mexE*, *acrD*, *mexE*, *(Bcr/CflA)*
2022GZP331	*AcrAB-TolC*, *AcrZ*, *norM*, *ompC*, *ompF*, *ompG*, *ompR*, *ompW*, *ompX*, *ompN_1*, *ompN_2*, *soxS*, *rob*, *marA*, *marB*, *marC*, *marR*, *emrA*, *emrB*, *emrE*, *mdfA*, *mexE*, *acrD*, *mexE*, *(Bcr/CflA)*
2022GZP221	*AcrAB-TolC*, *AcrZ*, *norM*, *ompC*, *ompF*, *ompG*, *ompR*, *ompW*, *ompX*, *ompN_1*, *ompN_2*, *soxS*, *rob*, *marA*, *marB*, *marC*, *marR*, *emrA*, *emrB*, *emrE*, *mdfA*, *mexE*, *acrD*, *mexE*, *(Bcr/CflA)*
2022GZP175	*AcrAB-TolC*, *AcrZ*, *norM*, *ompC*, *ompF_1*, *ompF_2*, *ompG*, *ompN*, *ompR*, *ompW*, *ompX*, *soxS*, *rob_1*, *rob_2*, *marA*, *marB*, *marC*, *marR*, *emrA*, *emrB*, *emrE*, *emrY*, *mdfA*, *mexE*, *acrD*, *mexE*, *(Bcr/CflA)*

Table S4. Primers used in this study.

Species	Target locus	Primer	Primer sequence (5’ to 3’)	Amplicon size	Reference
E. fergusonii	*lpxP*	EF-F	AGATTCACGTAAGCTGTTACCTT	575 bp	[52]
		EF-R	CGTCTGATGAAAGATTTGGGAAG		
E. coli	*cdgR*	EC-F	CCAGGCAAAGAGTTTATGTTGA	212 bp	[52]
		EC-R	GCTATTTCCTGCCGATAAGAGA		
	tet(X4)	*tet*(X4)-F	CTGATTCGTCTGACATCATCTTTTG	204 bp	[53]
		tet(X4)-R	GTTAAATTTCCCATTGGTCAGATTA		

52. Lindsey, R.L.; Garcia-Toledo, L.; Fasulo, D.; Gladney, L.M.; Strockbine, N. Multiplex polymerase chain reaction for identification of *Escherichia coli*, *Escherichia albertii* and *Escherichia fergusonii*. *J. Microbiol. Methods* **2017**, *140*, 1–4. https://doi.org/10.1016/j.mimet.2017.06.005.
53. Ji, K.; Xu, Y.; Sun, J.; Huang, M.; Jia, X.; Jiang, C.; Feng, Y. Harnessing efficient multiplex PCR methods to detect the expanding *tet*(X) family of tigecycline resistance genes. *Virulence* **2020**, *11*, 49–56. https://doi.org/10.1080/21505594.2019.1706913.
